# Adverse and Positive Childhood Experiences and Suicidal Ideation in US Children and Adolescents: A Systematic Review and Meta-Analysis

**DOI:** 10.7759/cureus.115899

**Published:** 2026-09-07

**Authors:** Dilyaver Matakhov, Shivani Shah, UFN Rizwanullah, Curie Villarson, Sharmin Ferdous, Michael Yassa, Chetachukwu Eze, Newton Rahming, Lauren L Wallace, Asher Masood, Syed Rahim, George Ampalloor, Opeyemi Alamu

**Affiliations:** 1 Family Medicine, Mehtercesme Family Medicine Center, Istanbul, TUR; 2 Occupational Health, Ekoglobal Occupational Health and Protection Services, Istanbul, TUR; 3 Medicine, Caribbean Medical University School of Medicine, Willemstad, CUW; 4 Internal Medicine, Hayatabad Medical Complex Peshawar, Peshawar, PAK; 5 Clinical Sciences, St. George's University School of Medicine, St. George's, GRD; 6 Medicine and Surgery, Rangpur Medical College and Hospital, Rangpur, BGD; 7 Medicine, St. George's University School of Medicine, St. George's, GRD; 8 Surgery, Caribbean Medical University, Willemstad, CUW; 9 Psychiatry, Lincolnwood Medical Center, Lincolnwood, USA; 10 Pediatrics, Humboldt Park Health, Chicago, USA; 11 Statistics, Federal College of Animal Health and Production Technology Ibadan, Ibadan, NGA

**Keywords:** adolescent, adverse childhood experiences, child, meta-analysis, positive childhood experiences, protective factors, suicidal ideation, systematic review

## Abstract

Suicide is a leading cause of premature death among US youth, and suicidal thoughts can emerge before adolescence. Adverse childhood experiences (ACEs) are established risk factors for suicidal ideation, while positive childhood experiences (PCEs) and related protective factors may reduce risk, yet no prior synthesis has quantified these associations across independent US pediatric and adolescent samples using effect estimates verified against their source publications. We searched PubMed/MEDLINE, PubMed Central, Centers for Disease Control and Prevention (CDC) publication databases, and reference lists for US-based studies that enrolled children or adolescents younger than 18 years, measured ACEs (cumulative or specific) and/or PCEs or analogous protective factors, reported self-reported suicidal ideation, and published a relative effect estimate with a 95% confidence interval (CI); one report per independent sample entered each pooled analysis. Estimates were pooled with DerSimonian-Laird random-effects models, risk of bias was assessed with the Newcastle-Ottawa Scale, and funnel-plot symmetry and Egger's test were examined for the primary pool but, with fewer than 10 studies, are reported only as exploratory. Eleven reports from 10 independent US samples met the criteria. In the primary meta-analysis of four independent samples, the highest ACE-exposure category (typically three to four or more ACEs) versus no ACEs was associated with substantially higher prevalence of suicidal ideation (pooled relative estimate: 7.19; 95% CI: 5.66-9.14; I-squared = 35%). A graded dose-response was evident, with pooled estimates of 2.78 (95% CI: 2.25-3.43) for the lowest ACE category and 4.60 (95% CI: 3.79-5.58) for intermediate categories, and in two studies reporting per-unit effects, each additional ACE was associated with 60% higher odds of suicidal ideation (pooled odds ratio: 1.60; 95% CI: 1.52-1.69; I-squared = 0%). Protective associations were consistent but not pooled: each additional PCE was associated with 13% lower risk of lifetime suicidal ideation at ages 11-12 (risk ratio: 0.87; 95% CI: 0.82-0.93), and parental monitoring, school connectedness, and family communication were associated with roughly 26-59% lower prevalence of ideation. Risk of bias was moderate for nine reports and high for two. Cumulative ACE exposure thus shows a strong, graded association with suicidal ideation among US children and adolescents, and protective childhood experiences and relationships are consistently associated with lower risk; because the pooled evidence is predominantly cross-sectional and adolescent, prospective preadolescent cohorts remain a priority, and these findings support combined risk- and strengths-based screening and prevention in pediatric and school settings.

## Introduction and background

Suicide remains a major public health concern among children and adolescents in the United States. Suicidal ideation, defined as thoughts about ending one's own life, is clinically important because it can emerge during childhood and often precedes suicide planning and attempts. Although suicidal ideation does not inevitably progress to suicidal behavior, its identification is essential for early prevention and intervention. These concerns highlight the importance of identifying modifiable risk and protective factors across childhood and adolescence. Curtin and Garnett [1] reported that the suicide rate among children aged 10-14 years tripled between 2007 and 2018, from 0.9 to 2.9 deaths per 100,000, and has remained elevated since. Suicidal thoughts and behaviors are not confined to older teenagers: Sheftall et al. [2] documented suicide deaths in elementary-school-aged children, with precipitating circumstances that differed from those of early adolescents. Concern intensified during the COVID-19 pandemic, when Yard et al. [3] found that emergency department visits for suspected suicide attempts rose among adolescents, particularly girls. Together, these trends make the identification of modifiable risk and protective factors across childhood and adolescence a public health priority.

Adverse childhood experiences (ACEs), defined as potentially traumatic events occurring before the age of 18 years, including abuse, neglect, and household dysfunction, are among the most consistently documented risk factors for poor mental health. The original ACE study by Felitti et al. [4] demonstrated a graded dose-response relationship between cumulative adversity and adult morbidity, and the meta-analysis by Hughes et al. [5] confirmed that exposure to multiple ACEs is strongly associated with mental illness, substance use, and self-directed violence across the life course. ACEs are common: Merrick et al. [6] estimated that most US adults report at least one and roughly one in six report four or more. For suicidality specifically, Angelakis et al. [7] pooled data across the lifespan and found that childhood maltreatment roughly doubled to tripled the odds of suicide attempts, though their synthesis was dominated by adult samples.

In contrast to this well-mapped risk, a growing literature examines protective experiences. Bethell et al. [8] showed that higher counts of positive childhood experiences (PCEs) were associated with better adult mental and relational health, even among adults reporting high ACE burden, and Crandall et al. [9] described "counter-ACEs" that appear to offset adversity. Evidence closer to the developmental window of interest comes from Janiri et al. [10], who used the Adolescent Brain Cognitive Development (ABCD) cohort to rank risk and protective factors for suicidality in US children aged 9-10 years, identifying family conflict as a risk factor and school engagement and parental monitoring as protective.

Despite this evidence, no meta-analysis has synthesized ACEs, PCEs, and suicidal ideation across independent US samples spanning childhood and adolescence using effect estimates verified against their source publications. Suicidal ideation in children younger than 13 years is studied far less often than in adolescents, so restricting a synthesis to the youngest ages alone leaves too few independent samples to pool; we therefore include US samples younger than 18 years and examine the preadolescent subset separately. Throughout, "children and adolescents" denotes participants younger than 18 years; where the preadolescent subset is discussed, ages are stated explicitly. This review set out to quantify the pooled association between cumulative ACE exposure and suicidal ideation in US children and adolescents, to synthesize the available evidence on PCEs and analogous protective factors, to assess the methodological quality of the contributing studies, and to identify the gaps that remain, particularly for preadolescents.

Materials and methods

Protocol and Reporting

This systematic review and meta-analysis was conducted and reported in accordance with the Preferred Reporting Items for Systematic Reviews and Meta-Analyses (PRISMA) 2020 statement [11]. The review was not prospectively registered. Every effect estimate was verified against the full text or the complete published tables of its source publication before extraction.

Eligibility Criteria

Studies were eligible when they analyzed a US sample of children or adolescents younger than 18 years; samples that also included some older participants were excluded, as were adult samples. Eligible studies had to measure ACEs, whether as a cumulative count or as defined specific adversities, and/or PCEs or analogous protective factors such as parental monitoring, school connectedness, or family communication. They also had to assess self-reported suicidal ideation, typically framed as having seriously considered attempting suicide in the past 12 months or over the lifetime, and to report an odds ratio (OR), a prevalence ratio (PR), or a risk ratio (RR) with a 95% confidence interval (CI). Policy statements, case series without a comparison group, non-US samples, and adult samples were excluded. Where a single cohort produced multiple eligible reports, one report per independent sample was retained for pooling; for the ABCD Study, which generated several relevant reports [10,12], a single report was kept, chosen for its later assessment wave and explicit PCE measurement (described in the Results).

Search Strategy and Data Extraction

Searches were conducted in 2026 using PubMed/MEDLINE, PubMed Central, and the Centers for Disease Control and Prevention (CDC) Morbidity and Mortality Weekly Report (MMWR), supplemented by forward and backward citation searching of retrieved reports and relevant reviews. Search terms combined ACEs and PCEs, suicidal ideation, and US child or adolescent populations. As summarized in the PRISMA 2020 flow diagram (Figure 1), 527 records were identified (486 from databases and registers, 41 from citation searching). After removing 73 duplicates, 454 records were screened, of which 396 were excluded. Fifty-eight reports were sought for retrieval, with three unavailable. Fifty-five full-text reports were assessed for eligibility, and 44 were excluded for predefined reasons. Eleven reports representing 10 independent samples met the inclusion criteria, with four independent samples contributing to the primary meta-analysis. For each included report, study characteristics, effect estimates, adjustment variables, and sample independence were extracted and verified against the full text or published tables.

**Figure 1:**
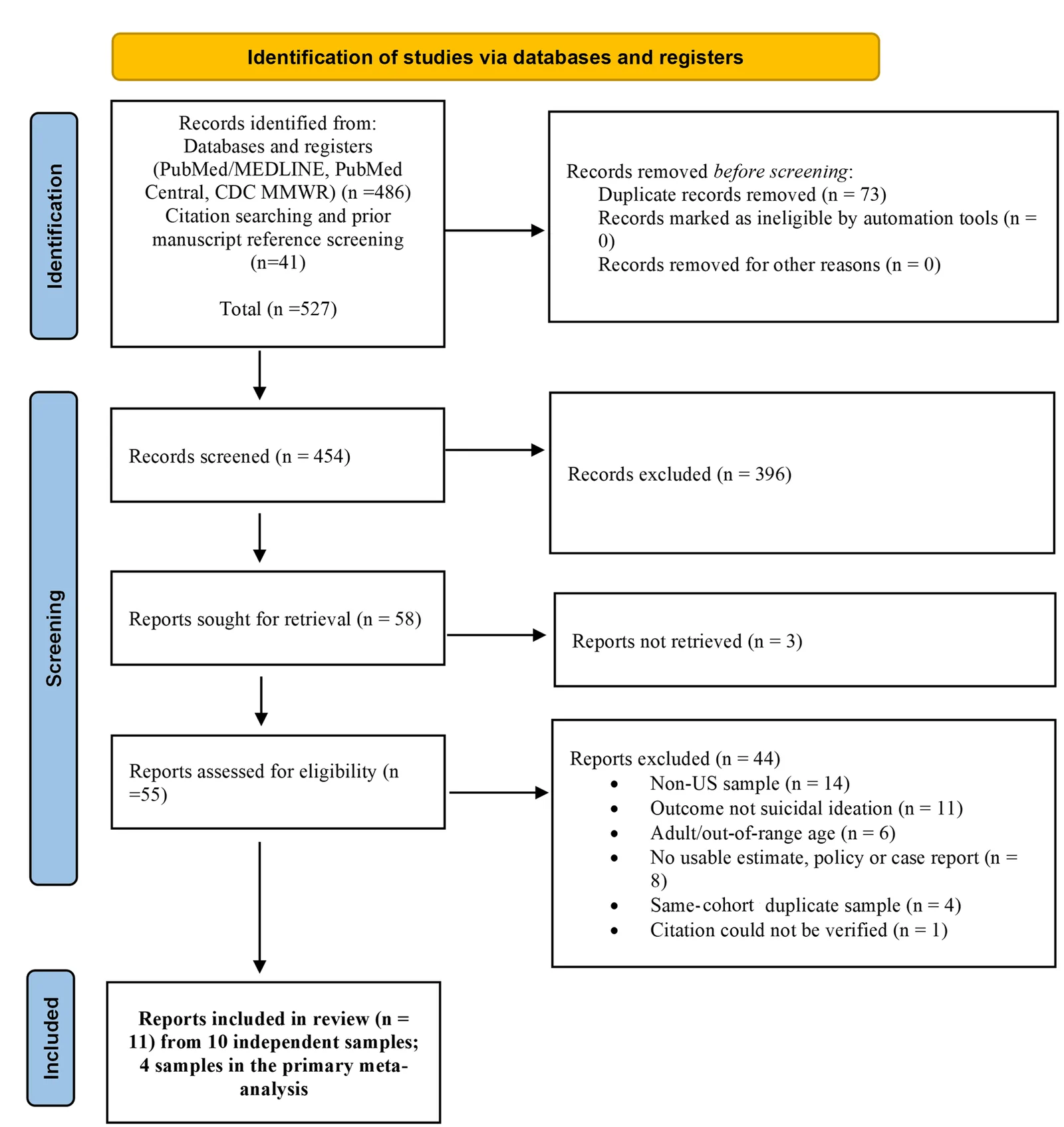
PRISMA 2020 flow diagram of the study selection. PRISMA: Preferred Reporting Items for Systematic Reviews and Meta-Analyses; CDC: Centers for Disease Control and Prevention; MMWR: Morbidity and Mortality Weekly Report

Risk of Bias

Methodological quality was assessed with the Newcastle-Ottawa Scale (NOS) [13] adapted for cross-sectional studies, scoring selection (0-4), comparability (0-2), and outcome ascertainment (0-3). Totals of at least 7 were classified as high quality, 4-6 as moderate, and 3 or below as low. Each study was scored on the three domains, and any discrepancies were resolved by discussion.

Statistical Analysis

Because included studies reported different exposure parameterizations, separate pools were pre-specified rather than forcing all estimates into one model. A primary pool compared the highest published ACE category (typically three to four or more ACEs) with no ACEs, a secondary pool combined per-one-unit ACE-count estimates, and further dose-response pools compared the lowest and intermediate ACE categories with none. Log-transformed estimates and standard errors derived from published CIs were pooled with DerSimonian-Laird random-effects models [14]; heterogeneity was quantified with Cochran's Q, the I-squared statistic [15], and the between-study variance tau-squared. Adjusted PRs and ORs were pooled together as relative measures; because suicidal ideation is not rare (commonly 15-25% in these samples), ORs exceed PRs for the same association, and this is flagged as a limitation. A leave-one-out sensitivity analysis was performed for the primary pool. Funnel-plot symmetry was examined visually, and Egger's regression test [16] was applied to the primary pool; because both assessments are underpowered with fewer than 10 studies [17], their results are reported as exploratory and are not used to draw firm conclusions about selective publication. PCE and protective-factor estimates were synthesized narratively because the underlying constructs are not a single exposure. Analyses were performed in R version 4.4 (R Foundation for Statistical Computing, Vienna, Austria) using the meta package with the metagen function and the DerSimonian-Laird estimator.

## Review

Study selection and characteristics

Eleven reports from 10 independent US samples met all criteria (Table 1): a prospective maltreatment-risk cohort reported by Thompson et al. [18]; an urban juvenile-detention intake sample [19]; a preadolescent foster-care sample [20]; the Nevada 2015 [21] and 2019 [22] Youth Risk Behavior Surveys (YRBS); the New Hampshire 2015 YRBS [23]; the national Adolescent Behaviors and Experiences Survey [24]; the ABCD cohort year-2 follow-up [25]; the national YRBS 2023 [26] with its protective-factor companion report [27]; and the Minnesota Student Survey [28]. Two samples assessed preadolescents directly (foster care, ages 9-11 [20]; ABCD, ages 11-12 [25]), and Thompson et al. [18] measured adversity from age 4 onward with outcome assessment at 16; the remaining samples were middle- and high-school aged.

**Table 1:** Characteristics of the included reports. Verlenden et al. [27] analyze the same YRBS 2023 sample as Swedo et al. [26] and was not entered as an independent sample in the ACE pool. ABCD: Adolescent Brain Cognitive Development; ABES: Adolescent Behaviors and Experiences Survey; FHF: Fostering Healthy Futures; HS: high school; LONGSCAN: Longitudinal Studies of Child Abuse and Neglect; PCE: positive childhood experience; YRBS: Youth Risk Behavior Survey; ACE: adverse childhood experience

Report	Design	Data source	Analytic (N)	Age/grade	Exposure(s)	Outcome
Thompson et al. [18]	Prospective cohort	LONGSCAN (5 US sites)	740	Exposure 4-16 y; outcome 16 y	8 adversity types	Ideation, past 12 mo
Bhatta et al. [19]	Cross-sectional	Juvenile detention intake	3,156	Mostly 12-17 y	4 adverse experiences	Lifetime ideation
Taussig et al. [20]	Cross-sectional (trial baseline)	Foster care (FHF trial)	515	9-11 y	Substantiated maltreatment types	Child-reported ideation
Clements-Nolle et al. [21]	Cross-sectional	Nevada YRBS 2015	4,955	Grades 9-12	5-item ACE score	Ideation, past 12 mo
Lensch et al. [22]	Cross-sectional	Nevada YRBS 2019	10,321	Middle + high school	ACE count; family communication; school connectedness	Suicidal behaviors incl. ideation
Aytur et al. [23]	Cross-sectional	New Hampshire YRBS 2015	10,603	Grades 9-12	8-item ACE score	Ideation, past 12 mo
Anderson et al. [24]	Cross-sectional	ABES 2021 (national)	4,390	HS, <18 y	7 pandemic-era ACEs	Ideation, past 12 mo
Bravo et al. [25]	Cross-sectional (cohort year 2)	ABCD Study	10,532	11-12 y	Parental legal-system involvement; PCE count	Lifetime ideation
Swedo et al. [26]	Cross-sectional	YRBS 2023 (national)	11,871	Grades 9-12, <18 y	8-item ACE count	Ideation, past 12 mo
Verlenden et al. [27]	Cross-sectional	YRBS 2023 (same sample)	20,103	Grades 9-12	Protective factors	Ideation, past 12 mo
Jewett et al. [28]	Cross-sectional (repeated)	Minnesota Student Survey 2013-2019	93,573	Grades 8, 9, 11	Structural ACEs	Ideation, past 12 mo

Risk of bias

Two reports were rated high quality (NOS total 7: [18] and [25]; both cohort designs with stronger comparability), and nine were rated moderate (NOS 5-6). No report was rated low quality (Table 2). The dominant limitations were same-source self-report of exposure and outcome and adjustment limited to demographic covariates in the school-survey studies.

**Table 2:** NOS quality assessment. NOS adapted for cross-sectional designs (selection 0-4; comparability 0-2; outcome 0-3; maximum 9). NOS: Newcastle-Ottawa Scale

Report	Selection (0-4)	Comparability (0-2)	Outcome (0-3)	Total (0-9)	Quality
Thompson et al. [18]	3	2	2	7	High
Bhatta et al. [19]	2	1	2	5	Moderate
Taussig et al. [20]	2	1	2	5	Moderate
Clements-Nolle et al. [21]	3	1	2	6	Moderate
Lensch et al. [22]	3	1	2	6	Moderate
Aytur et al. [23]	3	1	2	6	Moderate
Anderson et al. [24]	2	1	2	5	Moderate
Bravo et al. [25]	3	2	2	7	High
Swedo et al. [26]	3	1	2	6	Moderate
Verlenden et al. [27]	3	1	2	6	Moderate
Jewett et al. [28]	3	1	2	6	Moderate

Meta-analysis: cumulative ACEs and suicidal ideation

In the primary pool (highest ACE category versus none; four independent samples), adolescents in the highest published ACE category had substantially higher prevalence of suicidal ideation than those with no ACEs, with a pooled relative estimate of 7.19 (95% CI: 5.66-9.14; Z = 16.14; p < 0.001) and low-to-moderate heterogeneity (Q = 4.64; df = 3; I-squared = 35.3%; tau-squared = 0.021) (Figure 2). Study-level inputs were as follows: YRBS 2023, four or more ACEs, adjusted PR 9.15 (6.86-12.21) [26]; ABES 2021, four or more ACEs, adjusted PR 7.06 (5.02-9.93) [24]; Nevada 2015, three to five ACEs, adjusted OR 5.73 (4.11-7.99) [21]; and juvenile detention, all four adversities, adjusted OR 5.72 (2.02-16.20) [19].

**Figure 2:**
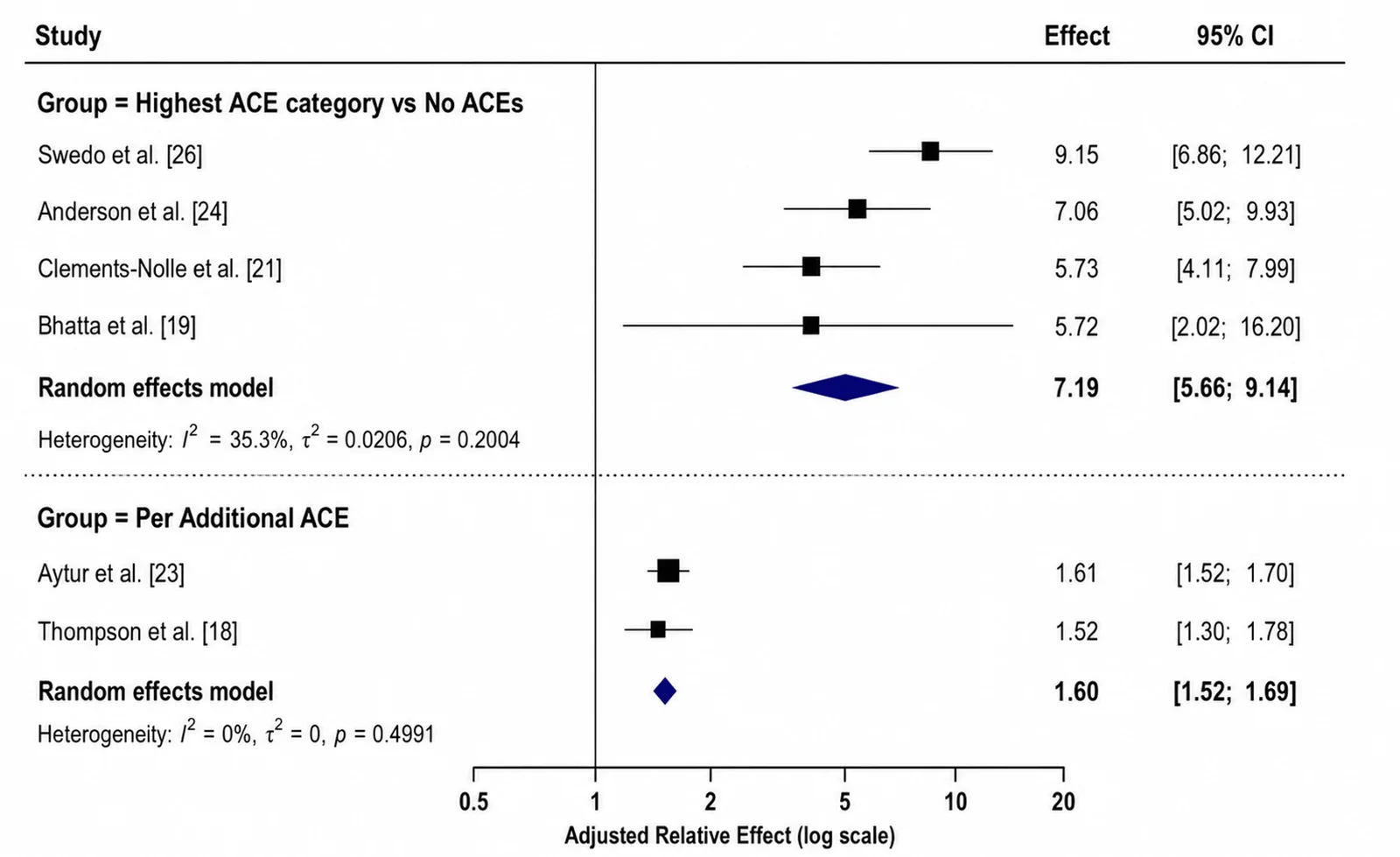
Forest plot of the primary pool (highest ACE category versus no ACEs) and the per-unit pool (per additional ACE). Squares are individual sample estimates with 95% confidence intervals; diamonds are DerSimonian-Laird random-effects pooled estimates. The x-axis is on a log scale. ACEs: adverse childhood experiences

A graded dose-response was evident across the three samples reporting full category sets [21,24,26]: pooled estimates were 2.78 (95% CI: 2.25-3.43; I-squared = 25.8%) for the lowest ACE category (one, or one to two, ACEs) and 4.60 (95% CI: 3.79-5.58; I-squared = 0.1%) for intermediate categories (two, two to three, or three ACEs), rising to 7.19 for the highest category. In the per-unit pool, each additional ACE was associated with 60% higher odds of suicidal ideation (pooled OR: 1.60; 95% CI: 1.52-1.69; I-squared = 0%), combining the New Hampshire YRBS (adjusted OR: 1.61; 1.52-1.70; [23]) and the prospective Longitudinal Studies of Child Abuse and Neglect (LONGSCAN) cohort (OR: 1.52; 1.30-1.78 per additional lifetime adversity; [18]). With only two studies, this pool is reported for consistency rather than precision.

The leave-one-out sensitivity analysis for the primary pool ranged from 6.31 (omitting [26]) to 8.09 (omitting [21]); no single sample changed the direction or statistical significance of the result. Several specific-adversity estimates were directionally consistent but parameterized differently and so were not pooled: physical abuse and suicidal ideation in foster-care preadolescents (OR: 1.9; 95% CI: 1.1-3.1; [20]); parental incarceration and lifetime ideation at ages 11-12 (RR: 1.74; 95% CI: 1.32-2.31; [25]); and individual structural ACEs and ideation across ethnoracial strata in Minnesota (ORs: approximately 1.18-1.51; [28]).

PCEs and protective factors

Protective estimates were synthesized narratively because constructs differed across studies. In the ABCD cohort at ages 11-12, each additional PCE was associated with 13% lower risk of lifetime suicidal ideation (RR: 0.87; 95% CI: 0.82-0.93), adjusted for ACE count and demographics; a formal PCE-by-parental-incarceration interaction was not statistically significant, so buffering in that cohort should be described as unproven [25]. In the national YRBS 2023, parental monitoring (adjusted PR range: 0.51-0.74 across levels), school connectedness (0.63-0.70), having a household adult who meets basic needs (0.41-0.80), and sufficient sleep (0.53-0.67) were associated with lower prevalence of seriously considering suicide [27]. In the Nevada 2019 YRBS, each one-unit increase in family communication or school connectedness was associated with 32-42% lower prevalence of suicidal behaviors among middle schoolers and 27-39% among high schoolers, with statistically supported buffering of the ACE association on the multiplicative scale [22]. Taken together, protective factors were associated with roughly 26-59% lower prevalence of suicidal ideation, with the strongest and most consistent signals for parental monitoring, family communication, and school connectedness.

Publication bias

For the primary pool, the funnel plot was approximately symmetric (Figure 3), and Egger's regression test did not detect small-study asymmetry (intercept -1.14; t = -0.56; df = 2; p = 0.63). With only four studies, however, this test is severely underpowered [16]: the estimated intercept is less precise than the asymmetry it is meant to detect. The result is therefore exploratory and cannot exclude selective publication, which is acknowledged as a limitation.

**Figure 3:**
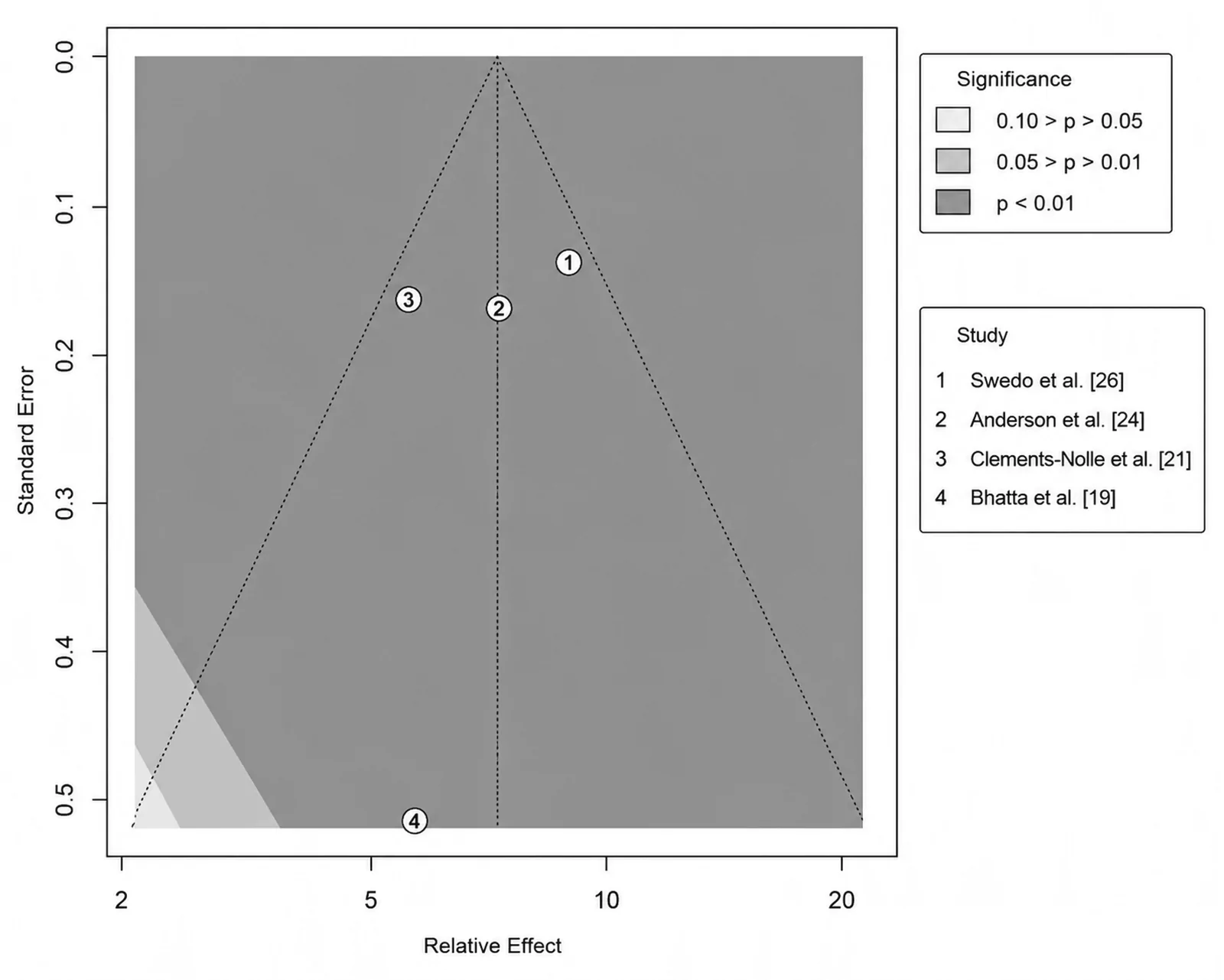
Funnel plot for the primary pool (highest ACE category versus no ACEs; four samples). Squares are individual sample estimates; the vertical line is the random-effects pooled estimate, and the dashed lines mark its pseudo-95% limits. With four studies, the plot is descriptive only, and formal asymmetry testing is underpowered. ACEs: adverse childhood experiences

Discussion

Across 10 independent US samples, cumulative ACE exposure showed a strong, graded association with suicidal ideation in children and adolescents. The pooled relative estimate of 7.19 for the highest exposure category versus none, the intermediate pooled estimates of 2.78 and 4.60, and the per-ACE pooled OR of 1.60 form a coherent dose-response pattern that is unlikely to be an artifact of any single study, as the leave-one-out analysis confirmed.

These results are consistent with, and somewhat stronger than, the lifespan meta-analysis of childhood maltreatment and suicidality by Angelakis et al. [7], which reported pooled ORs of roughly 2-3; the difference plausibly reflects the present contrast (highest category versus zero ACEs) and outcomes measured in adolescence, closer in time to the exposures. The overall direction aligns with the broader ACE-health synthesis of Hughes et al. [5]. The protective-factor findings are concordant with the adult PCE literature [8,9] and with ABCD-based reports that family-level factors relate to childhood suicidality [10,12]. Importantly, the one included study that formally tested an ACE-by-PCE interaction found none [25], whereas Lensch et al. [22] found buffering for family communication and school connectedness, so buffering should be described as construct-specific and not yet established for PCE counts.

Several mechanistic pathways may underlie these associations. Chronic early adversity is thought to dysregulate the stress-response system through allostatic load [29], with converging neurobiological and epidemiological evidence for enduring effects on brain development and emotion regulation [30,31]. Conversely, protective relationships may foster resilience, described by Masten [32] as arising from ordinary, modifiable developmental resources rather than rare traits, which is consistent with the buffering signals observed for family communication and school connectedness.

Clinically, the graded association supports ACE-informed suicide-risk awareness in pediatric and school settings, and the protective-factor results support strengths-based prevention. Promoting parental monitoring and communication and strengthening school connectedness, which Steiner et al. [33] linked to lower long-term suicidal ideation and other adult health harms, are actionable targets consistent with the scale of adolescent suicidal behavior documented by Nock et al. [34] and with the American Academy of Pediatrics trauma-informed care guidance [35]. The near-tenfold PR at four or more ACEs in the 2023 national YRBS [26] identifies a small, extremely high-risk group for whom screening and support are most urgent.

Limitations

Several limitations warrant emphasis. First, the pooled evidence is predominantly cross-sectional; temporality is established only in the LONGSCAN cohort [18], so the "onset" of ideation cannot be inferred and this term is avoided in the present claims. Second, because independent preadolescent samples are scarce, the eligibility band spans childhood and adolescence (younger than 18 years); the pooled results therefore describe mostly adolescents, and direct preadolescent evidence remains limited to three samples [18,20,25]. Third, adjusted PRs and ORs were pooled together; with outcome prevalences of 15-25%, ORs overstate PRs, so the primary pooled value should be read as a relative measure rather than an exact OR. Fourth, ACE instruments differed (four to eight items, some limited to past-12-month windows), as did ideation recall periods (12 months versus lifetime). Fifth, the review was not prospectively registered, and publication bias could be assessed only exploratorily given four studies in the primary pool, so selective publication cannot be excluded. Sixth, two included samples are special populations (detained youth; foster care), where both adversity and ideation are elevated.

## Conclusions

Cumulative ACE exposure is strongly and progressively associated with suicidal ideation among US children and adolescents: risk rises with each additional ACE (pooled OR: 1.60 per ACE) and is roughly sevenfold at the highest exposure levels relative to no ACEs. PCEs and protective relationships, particularly parental monitoring and communication and school connectedness, are consistently associated with substantially lower risk. Prospective, preadolescent, multi-cohort studies with harmonized ACE and PCE measurement remain the clearest research priority, and prevention should pair adversity-informed screening with the deliberate promotion of protective experiences.
